# Nitrite inhalants use, sexual behaviors and HIV/syphilis infection among men who have sex with men in Chongqing, China

**DOI:** 10.1186/s40249-020-00748-6

**Published:** 2020-09-04

**Authors:** Jin Chen, Yu-Ling Huang, Huai-Liang Chen, Ji Xia

**Affiliations:** 1 Department of Medical Records Management, the People’s Hospital of Tongliang District, Chongqing, China; 2 Department of STD/HIV Control and Prevention, Sichuan provincial Center for Disease Control and Prevention, Chengdu, China; 3 Department of Infectious Diseases Prevention and Healthcare, the People’s Hospital of Chengdu Tianfu New Area, Chengdu, China

**Keywords:** Nitrite inhalants use, Sexual behavior, HIV infection, Syphilis infection, Men who have sex with men

## Abstract

**Background:**

Emerging evidence indicates nitrite inhalants have become increasingly prevalent among men who have sex with men (MSM). The present study aimed to describe the prevalence and correlates of nitrite inhalants use and its association with risky sexual behaviors and human immunodeficiency virus (HIV)/syphilis infection among MSM in Chongqing, a city in China where MSM were burdened with the highest pooled HIV prevalence in the country.

**Methods:**

This cross-sectional study was conducted in Chongqing between March 2019 and February 2020. Information of demographics, drug use, sexual behaviors and HIV testing was collected through an anonymous survey. Blood samples were drawn from each participant for the diagnoses of HIV and syphilis. Logistic regression analysis was performed to evaluate factors correlated with nitrite inhalants use and its relationship with risky sexual behaviors and HIV/syphilis infection.

**Results:**

Of the 1151 eligible participants, 18.9% (218) reported use of at least one type of recreational drugs in the past 6 months, and nitrite inhalants were the most commonly used substance (17.7, 95% confidence interval [*CI*]: 15.6–20.2%). The proportions of participants reported engaging in group sex and practicing condomless internal ejaculation during anal sex in the past six months were 5.8% (95% *CI*: 4.4–7.2%) and 41.7% (95% *CI*: 38.7–44.7%), respectively. The general prevalence of HIV and syphilis infection among the enrolled MSM were 16.8% (95% *CI*: 14.7–19.0%) and 12.6% (95% *CI*: 10.7–14.4%), respectively. Factors positively associated with nitrite inhalants use included: age ≤ 25 (adjusted odds ratio [a*OR*] = 2.08, 95% *CI*: 1.10–3.94), monthly individual income ≥ CNY 3000 (Chinese Yuan) (a*OR* = 1.95, 95% *CI*: 1.18–3.22), preferring receptive anal intercourse (a*OR* = 2.27, 95% *CI*: 1.34–3.84) and versatile anal intercourse (a*OR* = 2.60, 95% *CI*: 1.64–4.13), age at first anal intercourse < 18 (a*OR* = 1.79, 95% *CI*: 1.21–2.67), engaging in group sex in the past six months (a*OR* = 9.34, 95% *CI*: 4.95–17.63), having multiple male sex partners in the past 6 months (a*OR* = 2.32, 95% *CI*: 1.50–3.58), practicing CIE during anal sex in the past six months (a*OR* = 1.71, 95% *CI*: 1.19–2.46), HIV infection (a*OR* = 1.72, 95% *CI*: 1.11–2.66) and syphilis infection (a*OR* = 1.98, 95% *CI*: 1.23–3.17).

**Conclusions:**

This study found that nitrite inhalants were the most commonly used recreational substance among MSM and nitrite inhalants use were associated with higher probability of HIV and syphilis infection. Therefore, increased attention and counselling should be given to nitrite inhalants-using MSM.

## Background

Despite the achievements obtained in human immunodeficiency virus (HIV)/acquired immune deficiency syndrome (AIDS) control and prevention in China, national updates on the AIDS/sexually transmitted diseases (STDs) epidemic indicated that men who have sex with men (MSM) were at increasing risk of HIV infection in the past decades. In 2017, 25.5% of the 134 512 newly diagnosed HIV cases were attributed to sex between men [1], while sexual contacts between men only accounted for 12.2% of the 50 000 new infections in 2007 [2]. The rapid spread of the HIV epidemic among MSM has posed great challenges to HIV/AIDS prevention in China.

Recent studies revealed that recreational drugs were increasingly popular among MSM in China [3–5]. Recreational drug use was reported to be associated with risky sexual behaviors, which placed users at elevated risk of HIV and STDs [5–7]. Previous studies reported that methamphetamine or ecstasy was the most prevalent recreational drug among MSM [8–10]. However, recent findings suggested nitrite inhalants (also referred to as rush poppers) had taken the place of methamphetamine or ecstasy and become the most popular recreational substance among MSM in China [11, 12].

Reports from western countries documented that MSM nitrite inhalants users were more likely to engage in risky sexual behaviors and were involved in greater likelihood of HIV acquisition [13, 14]. However, studies examining the associations between nitrite inhalants use and risky sexual behaviors, HIV infection and STDs are rather limited in China. Only a very small number of studies in north and east coastal metropolises of China (e.g., Beijing, Nanjing and Shenyang) addressed nitrite inhalants use among MSM and found nitrite inhalants use was significantly correlated with increased odds of HIV infection and engaging sexual behaviors, such as seeking male partners via the internet, having multiple male sex partners and practicing unprotected receptive anal intercourse [15–17].

Chongqing, the setting of the present study, is a provincial municipality located in the southwest of China. A systematic review of 355 studies covering 59 cities from 30 provinces and municipalities of China indicated MSM in Chongqing were inflicted with the highest pooled HIV prevalence (13.8%, 95% confidence interval [*CI*]: 12.8–14.9%, *N* = 24) in the country [18], whereas the prevalence of recreational drug use, especially nitrite inhalants use, among the subpopulation currently remained unknown. The present study aimed to describe the prevalence and correlates of nitrite inhalants use, as well as its associations with risky sexual behaviors and HIV/syphilis infection among MSM in this under-researched area, which could confirm and further extend the existing literature. Moreover, clarification of the issues might potentially contribute to the implementation of a more tailored and comprehensive HIV prevention strategy among MSM in China.

## Methods

### Study design and participants

This cross-sectional study was conducted from March 2019 to February 2020 in Chongqing. To be eligible to participate in the study, MSM had to be male, 18 years or older, have had sexual contact with men in the previous 12 months and were willing to provide written informed consent. The local community-based organization (CBO) covering the largest number of MSM was chosen as the recruitment site and MSM participants were recruited using the convenience sampling method.

Participants who provided written informed consent completed a self-administered anonymous survey in a designated room at the CBO. Each MSM participant was paid CNY 25 (Chinese Yuan) (about USD 4, United States Dollar) as remuneration. The protocol of this study was reviewed and approved by the Ethics Committee of the people’s hospital of Chongqing Tongliang District and the people’s hospital of Chengdu Tianfu New Area.

### Data collection

It took participants approximately 15 min to complete the paper-and-pencil questionnaire. The following information was collected: socio-demographic characteristics (including age, official residence location, education level, occupation, marital status and monthly individual income), types of drug used (including nitrite inhalants, capsule zero [5-MEO-DIPT, foxy], methamphetamine, ecstasy, magu [a kind of tablets which consist of methamphetamine and caffeine], ketamine, happy water [a mixture of crystal methamphetamine, ecstasy and ketamine], GHB [gamma-hydroxybutyrate], cannabis, bath salt [or cathinone], red crystal meth [extracts from methamphetamine] and heroin), sexual behaviors (including sexual orientation, preferred sexual role with males, age at first anal intercourse, route to seek male sex partners, unprotected anal intercourse [UAI] with regular partners and casual partners, number of male sex partners, group sex and condomless internal ejaculation [CIE] during anal sex in the past six months) and experience of HIV testing in the past six months.

### Laboratory testing

A 5-ml blood specimen was drawn from each participant to test their syphilis and HIV antibodies. For syphilis identification, the *Treponema pallidum* particle assay (TPPA) (Alere Medical Co., Ltd., Chiba Prefecture, Japan) was conducted, and cases with positive results were confirmed by the Toluidine red unheated serum test (TRUST) (Rongsheng Biotec, Shanghai, China). Participants who were tested positive in both TPPA and TRUST tests were considered as syphilis infection. For HIV evaluation, a rapid test (Determine HIV-1/2, Alere Medical Co., Ltd., Chiba Prefecture, Japan) and enzyme-linked immunosorbent assay (ELISA) (HIV Combo, Alere Medical Co., Ltd., Chiba Prefecture, Japan) were used to screen HIV antibodies, and positive cases were further confirmed by the western blot assay (HIV Blot 2.2 WB, MP Biomedicals Co., Ltd., Singapore) in the central lab of Chongqing Center for Disease Control and Prevention.

### Data analysis

Data were double entered using Epi Data software 3.1 (The Epi Data Association, Odense, Denmark) and analyzed using SPSS 16.0 (SPSS, Inc., Chicago, IL, USA) and SAS software 9.4 (SAS, Inc., Cary, NC, USA). Chi-square tests were used to compare the differences in socio-demographics, sexual behaviors, experience of HIV testing and HIV/syphilis infection between nitrite inhalants users and nonusers. Logistic regression analysis was employed to determine factors correlated with nitrite inhalants use. Variables with *P* < 0.05 in the univariate analysis were further entered into a multivariate stepwise logistic regression model and variables significant at *P* < 0.05 were retained in the final model. To examine the robustness of our results, Poisson regression model with robust standard error estimation was used to conduct sensitivity analysis, and prevalence ratios (PR) and 95% *CI* were obtained.

### Power analysis

The present analysis was part of a larger study which was designed to explore the prevalence of recreational drug use and its association with HIV/STDs among MSM in Chongqing. As no data on the prevalence of recreational drug use among MSM were reported before in this area, we referred to a recent survey from the neighboring Sichuan province reporting 27.7% of the MSM participants had ever used recreational drugs [5]. A sample size of 1044 was estimated based on an ability to detect changes in proportion of 10% from the estimated prevalence, with a power of 90% and a significance level of 5%. To obtain the estimated sample size, staff from the CBO introduced the study to the potential participants when they reached out in community to carry out health education activities and provided voluntary counseling and testing at the CBO. Enrolled participants were also encouraged to recommend their MSM peers.

## Results

### Characteristics of participants

In total, the CBO staff members managed to recruit 1162 potential participants and 11 (0.9%) MSM were excluded for declining to provide written informed consent. Thus, 1151 MSM were enrolled in the study. Of all the participants included, the average age was 30.44 years (standard deviation [*SD*] = 9.35), and about three-fourths (74.4%) of them officially resided in Chongqing city. Over two-thirds (68.9%) had obtained at least a college education degree, and most (79.5%) of the MSM participants were single. More than two-thirds (66.9%) reported to be service providers or solo business owners, and the majority (81.1%) reported a monthly individual income of CNY 3000 or more (Table 1).

**Table 1:** Socio-demographic characteristics among MSM by nitrite inhalants use status (*N* = 1151)

Characteristics	*n*	Percentage, 95% *CI*	Nitrite inhalants users (*n*, %)	Non–users (*n*, %)	*P* value
**Age (years), mean ±** ***SD***	30.44 ± 9.35		28.19 ± 6.28	30.9 ± 9.82	
≤ 25	410	35.6 (32.9–38.5)	82 (40.2)	328 (34.6)	0.001
26–35	491	42.7 (39.7–45.4)	98 (48.0)	393 (41.5)	
> 35	250	21.7 (19.5–24.2)	24 (11.8)	226 (23.9)	
**Residence in Chongqing**					
Yes	856	74.4 (71.9–76.8)	148 (72.5)	708 (74.8)	0.511
No	295	25.6 (23.2–28.1)	56 (27.5)	239 (25.2)	
**Education**					
Senior high school and below	358	31.1 (28.4–33.7)	64 (31.4)	294 (31.0)	0.927
College and above	793	68.9 (66.3–71.6)	140 (68.6)	653 (69.0)	
**Occupation**					
Enterprise, public institution or government	186	16.2 (14.1–18.2)	23 (11.3)	163 (17.2)	0.002
Service industry, solo business owner	770	66.9 (64.0–69.5)	158 (77.4)	612 (64.6)	
Retired, unemployed or student	195	16.9 (14.9–19.1)	23 (11.3)	172 (18.2)	
**Marital status**					
Never married	915	79.5 (77.0–81.8)	180 (88.2)	735 (77.6)	< 0.001
Married	162	14.1 (12.1–16.2)	11 (5.4)	151 (16.0)	
Divorced or widowed	74	6.4 (5.0–7.8)	13 (6.4)	61 (6.4)	
**Individual monthly income (CNY)**					
< 3000	217	18.9 (16.6–21.1)	25 (12.3)	192 (20.3)	0.008
≥ 3000	934	81.1 (78.9–83.4)	179 (87.7)	755 (79.7)	

*MSM* Men who have sex with men, *CI* Confidence interval, *SD* Standard deviation, *CNY* Chinese Yuan

Overall, most (66.7%) of the eligible participants claimed to be homosexual, and one-fifths (20.9%) of the participants reported their age at first anal intercourse was under 18 years old. The majority (67.2%) of them used gay applications to seek male sex partners, and over half (59.9%) reported having multiple sex partners in the previous six months. The proportion of MSM reporting UAI in the past six months with “regular sex partners” and with “casual sex partners” was 41.5 and 33.0%, respectively. 5.8% of the participants reported engaging in group sex, and over two-fifths (41.7%) practiced CIE during anal sex in the past six months. In this study, more than one-third (37.2%) of MSM had HIV testing experience in the past six months, and the laboratory test results showed that the general prevalence of HIV and syphilis infection among the enrolled MSM were 16.8% (95% *CI*: 14.7–19.0%) and 12.6% (95% *CI*: 10.7–14.4%), respectively (Table 2).

**Table 2:** Sexual behaviors and health outcomes among MSM by nitrite inhalants use status (*N* = 1151)

Characteristics	*n*	Percentage, 95% *CI*	Nitrite inhalants users (*n*, %)	Non–users (*n*, %)	*P* value
**Sexual orientation**					
Homosexual	768	66.7 (64.0–69.2)	167 (81.9)	601 (63.5)	< 0.001
Bisexual	320	27.8 (25.5–30.2)	35 (17.1)	285 (30.1)	
Uncertain	63	5.5 (4.3–6.9)	2 (1.0)	61 (6.4)	
**Age at first anal intercourse (years)**					
< 18	241	20.9 (18.5–23.3)	73 (35.8)	168 (17.7)	< 0.001
≥ 18	910	79.1 (76.7–81.5)	131 (64.2)	779 (82.3)	
**Seeking sex partners through gay applications in the past 6 months**					
Yes	774	67.2 (64.5–69.9)	166 (81.4)	608 (64.2)	< 0.001
No	377	32.8 (30.1–35.5)	38 (18.6)	339 (35.8)	
**Preferring sexual role with males**					
Insertive anal intercourse	383	33.3 (30.7–36.1)	33 (16.2)	350 (36.9)	< 0.001
Receptive anal intercourse	265	23.0 (20.7–25.4)	56 (27.4)	209 (22.1)	
Versatile anal intercourse	503	43.7 (41.0–46.6)	115 (56.4)	388 (41.0)	
**UAI with regular partners in the past 6 months**					
Yes	301	41.5 (37.7–44.9)	84 (55.6)	217 (37.7)	< 0.001
No	425	58.5 (55.1–62.3)	67 (44.4)	358 (62.3)	
**UAI with casual partners in the past 6 months**					
Yes	248	33.0 (29.7–36.3)	70 (44.0)	178 (30.0)	0.001
No	504	67.0 (63.7–70.3)	89 (56.0)	415 (70.0)	
**Number of male sex partners in the past 6 months**					
< 2	462	40.1 (37.4–42.9)	39 (19.1)	423 (44.7)	< 0.001
≥ 2	503	43.7 (40.9–46.6)	75 (36.8)	428 (45.2)	
≥ 5	186	16.2 (14.0–18.1)	90 (44.1)	96 (10.1)	
**Group sex in the past 6 months**					
Yes	67	5.8 (4.4–7.2)	49 (24.0)	18 (1.9)	< 0.001
No	1084	94.2 (92.8–95.6)	155 (76.0)	929 (98.1)	
**Condomless internal ejaculation during anal sex in the past 6 months**					
Yes	480	41.7 (38.7–44.7)	116 (56.9)	364 (38.4)	< 0.001
No	671	58.3 (55.3–61.3)	88 (43.1)	583 (61.6)	
**HIV testing in the past 6 months**					
Yes	428	37.2 (34.6–39.8)	91 (44.6)	337 (35.6)	0.016
No	723	62.8 (60.2–65.4)	113 (55.4)	610 (64.4)	
**HIV infection**					
Yes	193	16.8 (14.7–18.9)	55 (27.0)	138 (14.6)	< 0.001
No	958	83.2 (81.1–85.3)	149 (73.0)	809 (85.4)	
**Syphilis infection**					
Yes	145	12.6 (10.7–14.5)	44 (21.6)	101 (10.7)	< 0.001
No	1006	87.4 (85.5–89.3)	160 (78.4)	846 (89.3)	

*MSM* Men who have sex with men, *CI* Confidence interval, *UAI* Unprotected anal intercourse, *HIV* Human immunodeficiency virus

Among the 1151 participants in survey, 18.9% (218) reported having used at least one type of recreational drug in the past 6 months, of whom 20.6% (45) were polydrug users. Of all participants, 17.7% (204) reported use of nitrite inhalants in the past six months, followed by 4.5% (52) use of methamphetamine, 1.0% (12) use of magu, 0.3% (3) use of zero capsule, 0.3% (3) use of ketamine and 0.1% (1) use of cannabis, ecstasy, bath salt, red crystal meth and heroine, respectively. Compared with non-users, nitrite inhalants users were more likely to initiate anal intercourse at an earlier stage, seek sexual partners through gay applications, prefer receptive or versatile anal intercourse, practice sexual behaviors (including UAI with regular and casual sexual partners, multiple sex partners, group sex and CIE during anal sex), have experience of HIV testing and be HIV/syphilis positive (Table 2).

### Factors associated with nitrite inhalants use

Univariate analysis showed the following variables were positively associated with nitrite inhalants use (*P* < 0.05): age ≤ 25 years old, working in service industry, being solo business owners or others, being single, monthly individual income ≥ CNY 3000, preferring receptive or versatile anal intercourse, age at first anal intercourse < 18 years, seeking sex partners through gay applications, having multiple sex partners, engaging in group sex, practicing CIE during anal sex, having HIV testing experience in the past six months and HIV/syphilis infection (Table 3).

**Table 3:** Factors correlated with nitrite inhalants use in the past 6 months (*N* = 1151)

Factors	Univariate analysis		Multivariate analysis	
	*OR* (95% *CI*)	*P* value	a*OR* (95% *CI*)	*P* value
**Age (years)**				
> 25	1		1	
≤ 25	2.35 (1.50–3.69)	< 0.001	2.08 (1.10–3.94)	0.024
**Education**				
College and above	1			
Senior high school and below	1.02 (0.73–1.41)	0.927		
**Residence in Chongqing**				
Yes	1			
No	1.12 (0.80–1.58)	0.511		
**Occupation**				
Enterprise, public institution or government	1		1	
Service industry, solo business owners and others	1.64 (1.03–2.61)	0.038	1.32 (0.78–2.25)	0.305
**Marital status**				
Married, divorced or widowed	1		1	
Single	2.16 (1.38–3.40)	0.001	1.17 (0.61–2.22)	0.639
**Monthly individual income (CNY)**				
< 3000	1		1	
≥ 3000	1.82 (1.16–2.85)	0.009	1.95 (1.18–3.22)	0.009
**Preferring sexual role with males**				
Insertive anal intercourse	1		1	
Receptive anal intercourse	2.84 (1.79–4.52)	< 0.001	2.27 (1.34–3.84)	0.002
Versatile anal intercourse	3.14 (2.08–4.75)	< 0.001	2.60 (1.64–4.13)	< 0.001
**Age at first anal intercourse (years)**				
≥ 18	1		1	
< 18	2.58 (1.86–3.60)	< 0.001	1.79 (1.21–2.67)	0.004
**Seeking sex partners through gay applications in the past 6 months**				
No	1		1	
Yes	2.44 (1.67–3.55)	< 0.001	1.16 (0.74–1.82)	0.522
**Number of sex partners in the past 6 months**				
< 2	1		1	
≥ 2	3.42 (2.36–4.95)	< 0.001	2.32 (1.50–3.58)	< 0.001
**Group sex in the past 6 months**				
No	1		1	
Yes	16.32 (9.26–28.75)	< 0.001	9.34 (4.95–17.63)	< 0.001
**Condomless internal ejaculation during anal sex in the past 6 months**				
No	1		1	
Yes	2.11 (1.55–2.87)	< 0.001	1.71 (1.19–2.46)	0.004
**HIV testing in the past 6 months**				
No	1		1	
Yes	1.46 (1.07–1.98)	0.016	1.32 (0.93–1.89)	0.119
**HIV infection**				
No	1		1	
Yes	2.16 (1.51–3.10)	< 0.001	1.72 (1.11–2.66)	0.015
**Syphilis infection**				
No	1		1	
Yes	2.30 (1.56–3.41)	< 0.001	1.98 (1.23–3.17)	0.005

*OR* Odds ratio, *CI* Confidence interval, a*OR* Adjusted odds ratio, *HIV* Human immunodeficiency virus

In the final multivariate logistic regression model, nitrite inhalants use was significantly correlated with age ≤ 25 years old (odds ratio [*OR*] = 2.08, 95% *CI*: 1.10–3.94), monthly individual income ≥3000 CNY (*OR* = 1.95, 95% *CI*: 1.18–3.22), preferring receptive (*OR* = 2.27, 95% *CI*: 1.34–3.84) or versatile anal intercourse (*OR* = 2.60, 95% *CI*: 1.64–4.13), age at first anal intercourse < 18 years (*OR* = 1.79, 95% *CI*: 1.21–2.67), engaging in group sex in the past 6 months (*OR* = 9.34, 95% *CI*: 4.95–17.63), having multiple sex partners in the past six months (*OR* = 2.32, 95% *CI*: 1.50–3.58), practicing CIE during anal sex in the past six months (*OR* = 1.71, 95% *CI*: 1.19–2.46), HIV infection (*OR* = 1.72, 95% *CI*: 1.11–2.66) and syphilis infection (*OR* = 1.98, 95% *CI*: 1.23–3.17), (*P* < 0.05).

In the multivariate Poisson regression analysis, factors significantly associated with nitrite inhalants use were consistent with those in the multivariate logistic regression analysis (Table 4), namely: age ≤ 25 years old (PR = 1.04, 95% *CI*: 1.01–1.07), monthly individual income ≥ 3000 CNY (PR = 1.04, 95% *CI*: 1.01–1.09), preferring receptive (PR = 1.04, 95% *CI*: 1.01–1.07) or versatile anal intercourse (PR = 1.05, 95% *CI*: 1.03–1.08), age at first anal intercourse < 18 years (PR = 1.05, 95% *CI*: 1.02–1.09), engaging in group sex in the past 6 months (PR = 1.37, 95% *CI*: 1.26–1.49), having multiple sex partners in the past six months (PR = 1.05, 95% *CI*: 1.03–1.07), practicing CIE during anal sex in the past six months (PR = 1.03, 95% *CI*: 1.01–1.06), HIV infection (PR = 1.05, 95% *CI*: 1.01–1.09) and syphilis infection (PR = 1.06, 95% *CI*: 1.02–1.10), (*P* < 0.05).

**Table 4:** Poisson regression analysis of factors correlated with nitrite inhalants use in the past 6 months (*N* = 1151)

Factors	Multivariate analysis	
	Prevalence ratio (95% *CI*)	*P* value
**Age (years)**		
> 25	1	
≤ 25	1.04 (1.01–1.07)	0.025
**Occupation**		
Enterprise, public institution or government	1	
Service industry, solo business owners and others	1.01 (0.99–1.04)	0.367
**Marital status**		
Married, divorced or widowed	1	
Single	1.01 (0.98–1.04)	0.602
**Monthly individual income (CNY)**		
< 3000	1	
≥ 3000	1.04 (1.01–1.09)	0.003
**Preferring sexual role with males**		
Insertive anal intercourse	1	
Receptive anal intercourse	1.04 (1.01–1.07)	0.004
Versatile anal intercourse	1.05 (1.03–1.08)	< 0.001
**Age at first anal intercourse (years)**		
≥ 18	1	
< 18	1.05 (1.02–1.09)	0.003
**Seeking sex partners through gay applications in the past 6 months**		
No	1	
Yes	1.01 (0.99–1.03)	0.46
**Number of sex partners in the past 6 months**		
< 2	1	
≥ 2	1.05 (1.03–1.07)	< 0.001
**Group sex in the past 6 months**		
No	1	
Yes	1.37 (1.26–1.49)	< 0.001
**Condomless internal ejaculation during anal sex in the past 6 months**		
No	1	
Yes	1.03 (1.01–1.06)	0.008
**HIV testing in the past 6 months**		
No	1	
Yes	1.02 (1.00–1.05)	0.065
**HIV infection**		
No	1	
Yes	1.05 (1.01–1.09)	0.018
**Syphilis infection**		
No	1	
Yes	1.06 (1.02–1.10)	0.007

*CI* Confidence interval, *HIV* Human immunodeficiency virus

## Discussion

The present study explored the prevalence and correlates of nitrite inhalants use and its association with risky sexual behaviors and HIV/syphilis infection among a sample of community-based MSM. Corroborating previous findings [5, 6, 17], nitrite inhalants were found to be the most commonly used recreational substance among MSM in Chongqing. Of the 1151 participants included, 204 (17.7%) reported nitrite inhalants use in the past 6 months, which was higher than 3-month use rate (10.6%) in Shenzhen [12], but lower than reports from metropolises elsewhere in China, such as Shenyang (18.9%) [15], Beijing and Nanjing (29.8%) [17] and life-time use rate (24.1%) in three cities of Sichuan Province [19]. The reason why nitrite inhalants have outnumbered other illicit drugs and risen in popularity might be attributed to its function on sexual pleasure enhancement, easy accessibility via the internet and not being included in the list of illegal drugs.

Our study found that MSM with younger age and higher monthly individual income were more likely to be nitrite inhalants users, which was consistent with results in previous studies [15, 16]. Younger MSM may be more familiar with the internet and have better access to nitrite inhalants. Meanwhile, nitrite inhalants use might be quite an expenditure to lower-income MSM. Therefore, MSM with such demographic characteristics as younger age and higher income were more likely to use nitrite inhalants than their counterparts. Additionally, several studies have demonstrated that MSM preferring receptive anal intercourse or versatile anal intercourse were more likely to be nitrite inhalants users [12, 16], and a similar result was found in this study. One possible explanation could be that nitrite inhalants have the effect of relaxing the anal sphincter, which could prevent injury and facilitate sex by not causing pain during anal penetration [20, 21]. Furthermore, we found that earlier anal sex experience was correlated with nitrite inhalants use. Similar to findings in this study, prior studies indicated that MSM with earlier anal sex initiation were more likely to engage in risky behaviors, such as substance use, in their later life [22, 23]. Thus, close attention should be given to MSM with characteristics mentioned above, and these subgroups should be a priority for further interventions towards drug use.

Evidence from specific countries revealed nitrite inhalants use was significantly associated with risky sexual behaviors [13, 24, 25]. However, only a small number of studies in China reported the association between nitrite inhalants use and sexual behaviors, like seeking male sexual partners via the internet, having multiple sex partners and practicing UAI [16, 17, 26]. In the present study, we confirmed the association between nitrite inhalants use and multiple sex partnerships. Additionally, we found nitrite inhalants users were more likely to engage in group sex and practice CIE during anal sex in the past 6 months. It was previously reported that MSM commonly use nitrite inhalants or erectile dysfunction medications before or during group sex [25, 27–29]. Nitrite inhalants were known to increase sexual desire, maintain erection and enhance sexual pleasure [16, 21], which might partly explain why MSM were more likely to use nitrite inhalants in the context of group sex. Moreover, similar to other recreational drugs, nitrite inhalants may also affect central nervous system function and influence decision-making [30, 31]. Thus, it is unsurprising that MSM who use nitrite inhalants practice higher levels of CIE during anal sex.

In accordance with prior reports [12, 17], our data indicated that MSM who used nitrite inhalants in the past 6 months were, respectively 1.72 times and 1.98 times, as likely to acquire HIV and syphilis as nonusers. The existing literature [12, 16, 19], as well as findings in this study, demonstrated that nitrite inhalants use was significantly associated with sexual behaviors, such as having multiple sex partners, engaging in group sex, and practicing UAI and CIE, which may contribute to increased odds of HIV and syphilis infection. In addition, we found high prevalence of risky sexual behaviors, HIV and syphilis among both nitrite inhalants-using MSM (27.0% for HIV and 21.6% for syphilis) and nonusers (14.6% for HIV and 10.7% for syphilis), which suggested comprehensive measures were needed to prevent transmission of HIV/STDs among MSM in China.

Several potential limitations should be addressed in this study. Firstly, the evidence for causal relationships between nitrite inhalants use and risky sexual behaviors and HIV/syphilis infection is weak due to the cross-sectional design of the study. Secondly, participants in this study consisted of MSM who volunteered to attend a CBO. Given the fact that not all MSM would be willing to go to a CBO, and that the CBO attendees might be different from those who were not willing to attend a CBO, our study may be subjected to selection bias. Furthermore, the study collected sensitive information on monthly income, drug use and sexual behaviors, and social desirability bias might exist. In order to reduce the respondents’ anxiety and reluctance, the purpose of the study was fully explained before the survey started and anonymous questionnaire was administered in private rooms. Lastly, the results were based on a self-administered questionnaire survey and might be influenced by recall bias.

## Conclusions

This study found nitrite inhalants were commonly used among MSM in Chongqing and MSM who reported nitrite inhalants use were more likely to practice risky sexual behaviors and were at higher probability of HIV and syphilis infection than their counterparts. The findings suggested particular attention and counselling should be given to nitrite inhalants-using MSM and integrate strategies should be tailored to mitigate nitrite inhalants use and its potential risks.

## Data Availability

The datasets used during the current study are available from the corresponding author on reasonable request.

## Abbreviations

MSM: Men who have sex with men
HIV: Human immunodeficiency virus
STDs: Sexually transmitted diseases
AIDS: Acquired immune deficiency syndrome
CIE: Condomless internal ejaculation
UAI: Unprotected anal intercourse
TPPA: Treponema pallidum particle assay
TRUST: Toluidine red unheated serum test
ELISA: Enzyme-linked immunosorbent assay
*OR*: Odds ratio
*CI*: Confidence interval
a*OR*: Adjusted odds ratio
*SD*: Standard deviation
PR: Prevalence ratios
CBO: Community-based organization
CDC: Center for Disease Control and Prevention
